# Disease features and outcomes among US lupus patients of Hispanic origin and their Mestizo counterpart in Latina America

**DOI:** 10.1186/ar4649

**Published:** 2014-09-18

**Authors:** Manuel Ugarte-Gil, Guillermo J Pons-Estel, Daniel Wojdyla, Gerald McGwin, Bernardo A Pons-Estel, Graciela S Alarcón

**Affiliations:** 1 Universidad Cieantífica del Sur and Hospital Nacional Guillermo Almenara Irigoyen, Lima, Perú; 2 Institut Clínic de Medicina i Dermatología, Hospital Clínic, Barcelona, Spain; 3 Escuela de Estadística, Universidad Nacional de Rosario, Rosario, Argentina; 4 The University of Alabama at Birmingham, Birmingham, AL, USA; 5 Hospital Provincial de Rosario, Rosario, Argentina

## Background

Systemic lupus erythematosus (SLE) US Hispanic patients with a large Amerindian ancestral background have been found to have poor outcomes. Similar observations have been made in the mixed population of Latin America. We are now comparing and contrasting selected sociodemographic and clinical features and outcomes of lupus patients from these two groups.

## Methods

SLE US Hispanic patients (European and Amerindian ancestry) from the LUMINA cohort (Lupus in Minorities: Nature vs. Nurture) and Latin American Mestizo patients from the GLADEL cohort (Grupo LatinoAmericano De Estudio de Lupus (Latin American Group for the Study of Lupus)) constitute the study population. Only patients who fulfilled four of the 1997 ACR criteria were included. Diagnosis time was time to the fourth criterion. Demographic and clinical data from these patients were extracted. When the ascertainment method for a specific variable was different in both cohorts, this has been noted. All variables were then compared using descriptive statistical tests. Adjustment for disease duration was done when indicated using either a Poisson regression or logistic regression, as appropriate.

## Results

Salient features for these two patient groups are presented in Table 1. Some of the differences observed in terms of the socioeconomic features could be due to the different methods of ascertainment.

**Table 1:** Salient features of US Hispanic SLE patients from LUMINA and Latin America Mestizo patients from GLADEL (at diagnosis or at last visit)

Characteristic	LUMINA (*n *= 114)	GLADEL (*n *= 619)	*P *value
Age, mean (SD)	31.3 (12.2)	29.4 (12.6)	0.138
Gender (female), *n *(%)	106 (93.0)	546 (88.2)	0.135
Disease duration (years), mean (SD)	6.1 (4.3)	4.5 (4.6)	<0.001
Low SES^a^, *n *(%)	42/107 (39.3)	391 (63.2)	<0.001
Health insurance, *n *(%)	56/112 (50.0)	466/615 (72.5)	<0.001
Acute onset, *n *(%)	34 (29.8)	151 (24.4)	0.220
ACR criteria number^b^, mean (SD)	6.8 (1.6)	6.3 (1.5)	0.069
Disease activity (moderate-high)^c^, *n *(%)	78/92 (84.8)	438/493 (88.8)	0.268
Renal disorder, *n *(%)	60 (52.6)	370 (59.8)	0.155
SDI score at last visit^b^, mean (SD)	2.3 (2.6)	1.7 (1.7)	<0.001
Renal damage (per SDI, at last visit)^d^, *n *(%)	37 (32.5)	184 (29.7)	0.559
Deceased^d^, *n *(%)	21 (18.4)	35 (5.7)	<0.001

SES, socioeconomic status; ACR, American College of Rheumatology; SDI, SLICC (Systemic Lupus International Collaborating Clinics) Damage Index. ^a^SES defined as being below the Federally-defined poverty line for LUMINA and as per the Graffar method for GLADEL. ^b^After adjusting for disease duration in a Poisson regression model. ^c^Defined as a SLAM (Systemic Lupus Activity Measure) >7 for LUMINA and a SLEDAI (Systemic Lupus Erythematosus Disease Activity Index) >4 for GLADEL. ^d^Significant after adjusting for disease duration using a Poisson regression model (for the SDI) and logistic regression for mortality

## Conclusions

Patients in both cohorts exhibited active disease, with renal involvement and damage being frequent, and overall damage accruing rapidly; however, the US Hispanic patients exhibited a higher mortality. These two patient groups were also of low SES. These data suggest that these two populations share an underlying genetic background which coupled with a poor SES places them at increased risk for severe lupus with unfavorable short, intermediate and long-term outcomes. The less favorable mortality experience of the US Hispanics deserves to be further examined.

